# Hallmarks in prostate cancer imaging with Ga68-PSMA-11-PET/CT with reference to detection limits and quantitative properties

**DOI:** 10.1186/s13550-018-0378-4

**Published:** 2018-04-04

**Authors:** Alejandro Sanchez-Crespo, Emma Jussing, Ann-Charlotte Björklund, Katja Pokrovskaja Tamm

**Affiliations:** 1 Department of Oncology-Pathology, Karolinska Institutet, Stockholm, Sweden; 2 Department of Hospital Physics and Nuclear Medicine, Karolinska University Hospital, Stockholm, Sweden; 3 Department of Radiopharmacy, Karolinska University Hospital, Stockholm, Sweden; 4 Department of Clinical Neuroscience, Karolinska Institutet, Stockholm, Sweden

**Keywords:** Prostate cancer, PSMA, PET, Detection limits, Image quantification

## Abstract

**Background:**

Gallium-68-labeled prostate-specific antigen positron emission tomography/computed tomography imaging (Ga68-PSMA-11-PET/CT) has emerged as a potential gold standard for prostate cancer (PCa) diagnosis. However, the imaging limitations of this technique at the early state of PCa recurrence/metastatic spread are still not well characterized. The aim of this study was to determine the quantitative properties and the fundamental imaging limits of Ga68-PSMA-11-PET/CT in localizing small PCa cell deposits.

**Methods:**

The human PCa LNCaP cells (PSMA expressing) were grown and collected as single cell suspension or as 3D-spheroids at different cell numbers and incubated with Ga68-PSMA-11. Thereafter, human HCT116 cells (PSMA negative) were added to a total cell number of 2 × 10^5^ cells per tube. The tubes were then pelleted and the supernatant aspirated. A whole-body PET/CT scanner with a clinical routine protocol was used for imaging the pellets inside of a cylindrical water phantom with increasing amounts of background activity. The actual activity bound to the cells was also measured in an automatic gamma counter. Imaging detection limits and activity recovery coefficients as a function of LNCaP cell number were obtained. The effect of Ga68-PSMA-11 mass concentration on cell binding was also investigated in samples of LnCaP cells incubated with increasing concentrations of radioligand.

**Results:**

A total of 1 × 10^4^ LNCaP cells mixed in a pellet of 2 × 10^5^ cells were required to reach a 50% detection probability with Ga68-PSMA-11-PET/CT without background. With a background level of 1 kBq/ml, between 4 × 10^5^ and 1 × 10^6^ cells are required. The radioligand equilibrium dissociation constant was 27.05 nM, indicating high binding affinity. Hence, the specific activity of the radioligand has a profound effect on image quantification.

**Conclusions:**

Ga68-PSMA-11-PET detects a small number of LNCaP cells even when they are mixed in a population of non-PSMA expressing cells and in the presence of background. The obtained image detection limits and characteristic quantification properties of Ga68-PSMA-11-PET/CT are essential hallmarks for the individualization of patient management. The use of the standardized uptake value for Ga68-PSMA-11-PET/CT image quantification should be precluded.

## Background

Prostate cancer (PCa) patients may develop local recurrences and metastatic spread after primary treatment. A general indicator of possible relapse is an increasing concentration of prostate-specific antigen (PSA) in the blood. PSA is generally regarded as a good biomarker for disease progression in primary PCa, and it is a well-studied surrogate of tumor burden [1–4]. However, at an early PCa recurrence or microscopic metastatic spread, PSA alone can seldom be used for staging and is generally associated with relatively low levels of serum PSA concentrations (< 0.2 ng/ml). If the PCa recurrence is diagnosed at an early stage, the patient can still be treated with curative intentions. A recent study showed that patients with biochemical PCa recurrence and treated with salvage radiation therapy (SRT) have a long term disease-free rate which rapidly declines with increasing levels of PSA at treatment start [5]. Further, another study showed that about half of the PCa patients treated with SRT are likely to suffer again a biochemical failure [6]. These adverse therapeutics results may correspond to patients with disseminated disease, which should have been treated with systemic therapy instead of—or in combination with—SRT, which is more successful for focal recurrences. These patients may even become more symptomatic after SRT. Hence, the proper imaging diagnostic tool to discriminate patients with local recurrence from advanced metastatic PCa is essential to select the most adequate treatment strategy based on the spread and anatomical location of the disease. However, to date, there is no gold standard diagnostic imaging technique for this patient group. At low PSA levels, conventional imaging diagnostic techniques, like ultrasound, magnetic resonance, and computed tomography, have shown limitations at discriminating patients with local recurrence from those with disseminated disease [7]. Deep biopsies are, on the other hand, difficult to correlate with imaging, have difficult histological interpretation and need skills to be successfully accomplished [8, 9]. Recently, positron emission tomography/computer tomography (PET/CT) imaging of the Gallium-68-labeled prostate-specific membrane antigen (Ga68-PSMA) has emerged as a potential gold standard to monitor PCa recurrence/metastatic spread even at low PSA levels [10, 11]. The literature on the topic revealed that, from all image modalities, Ga68-PSMA-PET/CT is to date the most accurate test for patient follow-up and staging. Ga68-PSMA-PET/CT outperforms also the detection rates of other PET tracers commonly used for PCa-like C11-Acetate and C11-Choline, even at low PSA [12]. Ga68-PSMA-PET/CT images have recently been used to deliver SRT with dose escalation to focal and solitary Ga68-PSMA uptakes in the prostate bed and pelvic nodes [13]. In such approach, good knowledge on the accuracy of Ga68-PSMA-PET to establish micro-metastatic involvement is therefore essential for patient selection. Still, there is no clear consensus on the limitations of this technique [14]. This may be due to the lack of a gold standard to which it can be compared against at low PSA and a lack of studies on the limitations of the Ga68-PSMA-PET/CT-imaging technique at microscopic stage. Modern PET cameras have high sensitivity, but at very low cellular concentration levels, the limiting physical factors inherent to the PET technique [15–17] as well as the specific biological and pharmacological properties of the Ga68-PSMA tracer hamper tumor imaging performance. Phantom experiments characterizing PET tracer detectability are generally useful only to characterize one or a few of these factors at a time. Put together, phantom-derived PET scanner performance, in terms of minimum detectable activity for Ga68-PSMA tracer, may not reflect the true in vivo realization. The aim of this study was to determine the fundamental limits of Ga68-PSMA-PET/CT in localizing small prostate cancer cell deposits, by taking into consideration the summative effects of the physical, biological, and pharmacological factors in a single set of experiments. Additionally, the quantitative properties of PCa imaging with Ga68-PSMA-PET/CT were also investigated.

## Methods

### Preparation of the Ga68-PSMA-11 radioligand

In this work, the radioligand [Ga68]-PSMA-HBED-CC (Ga68-PSMA-11) was used [18]. Briefly, the PSMA-11 precursor (ABX, Radeberg, Germany) was labeled using Ga(68)Cl_3_ eluted from a Gallia Pharm Ge68/Ga68-generator (Eckert & Ziegler, Berlin, Germany) in a cassette-based automated synthesis module ModularLab PharmTracer (Eckert & Ziegler, Berlin, Germany). High-performance liquid chromatography (HPLC; Shimadzu, Kyoto, Japan) was used to determine the initial specific radioactivity (SRA). Analyses for radiochemical purity were performed by HPLC and by instant thin layer chromatography.

### PET/CT system and image acquisition protocol

A Discovery 710 PET/CT whole-body system (General Electric Medical Systems, Milwaukee, WI, USA) was used throughout this work (3D-sensitivity of 7.9 cps/kBq and approximately 5 mm spatial resolution at the center of the field of view) with a routine clinical image acquisition protocol of 3 min per bed, 192 × 192 pixels matrix size and 0.043 cm^3^ voxel. All PET images were reconstructed using OSEM with time-of-flight and CT-based attenuation correction.

### Cell cultures

The PSMA expressing human prostate cancer cell line LNCaP and the PSMA-negative human colorectal carcinoma cell line HCT116, from the American Type Culture Collection, were used in this work. Cell lines were cultured in RPMI 1640 medium (GE Healthcare HyClone, USA, cat no# SH30096.01) supplemented with 10% fetal calf serum (Gibco by Thermo Fisher Scientific, MA-USA, cat no# 10270-106), 1% l-glutamine (Gibco by Thermo Fisher Scientific, MA-USA, cat no# 25030-024), and 1% PEST (GIBCO Penicillium-Streptomycin, Thermo Fisher Scientific, MA-USA, no# 15140-122) in 5% CO_2_ humidified air at 37 °C. Thereafter, each cell line was trypsinized (GIBCO Trypsin EDTA 0.05%, phenol red, Thermo Fisher Scientific, MA-USA, cat no# 25300-096) in a single cell suspension. Cell number and PSMA expression were measured using flow cytometry (FACS) with a BD-LSR II Flow Cytometer (BD Biosciences, USA) and Alexa Fluor 488 anti-human PSMA antibody (BioLegends, San Diego, USA, cat no# 342506) for cell staining.

### Ga68-PSMA-11-PET/CT detection limits and image quantification

To investigate the limitation of Ga*68-PSMA-11-PET/CT* to detect small amounts of LNCaP cells, an experimental setup including physical and biological properties was devised. LNCaP cells were first grown in three-dimensional spheroids from a suspension of single cells [19]. In brief, an initial suspension of approximately 2500 LNCaP cells were seeded using sterile-filtered (Acrodisc 25 mm Syringe Filter w/0.2 μm with Supor membrane, PALL corporation, New York, USA) RPMI 1640 media complete in each of the wells of an ultra-low attachment, poly-HEMA-coated round bottom 96-well plate (Corning life Sciences, MA, USA, cat no# CLS3474). The plates were inverted to allow cell sedimentation during 24 h. Thereafter, the plates were flipped back and incubated for 5 days. The number of living LNCaP cells and PSMA expression in the spheres, stained with Alexa Fluor 488 anti-human PSMA antibody, were measured using FACS. A total of 40 spheroids were obtained from the 96-well plates and placed in a 1.5-ml Eppendorf tube together with RPMI 1640 cell culture media to a total volume of 1 ml. The same procedure was repeated in samples of 20, 10, 5, 2, and 1 spheroids. A total of 500 kBq of Ga68-PSMA-11 was added to each tube. Thereafter, the tubes were incubated at 37 °C during 30 min.

### Supernatant removal

At the end of the incubation time, the tubes were centrifuged at 2780 rpm for 3 min, after adding single HCT116 cells (PSMA negative) to a concentration of approximately 2 × 10^5^ LNCaP+HCT116 cells in each tube. This allows the formation of a visible white color pellet (located down the tip of the tubes) of equal size (approximately 1 mm diameter) but with different numbers of LNCaP cells in each tube. The supernatant containing the excess of Ga68-PSMA-11 together with the incubation media was then carefully aspirated. Thereafter, each pellet was washed twice with cold phosphate buffered saline (PBS; HyClone DPBS/MODIFIED cat no# SH30378.02). Finally, 1 ml of fresh PBS was added to each tube. The tubes were then placed inside of a 5-l cylindrical water phantom and scanned in the whole-body PET/CT camera. This experiment was repeated at four independent occasions.

To include another biological configuration to this assay, the experiment was repeated incubating a total of 1 × 10^5^, 5 × 10^4^, 2.5 × 10^4^, 1.2 × 10^4^, 6 × 10^3^, and 3 × 10^3^ samples of single LNCaP cells in suspension with the radioligand for 30 min. This assay was performed in duplicate at four different occasions.

In real patients, also PSMA showed a high specificity, background activity, especially close to the prostate may be present. Background level depends on many different factors like patient compliance (for instance in terms of hydration), the use of diuretics prior the PET scan, the tracer SRA, and the image acquisition protocol. This generally results in inhomogeneous background distribution. To account for this phenomenon, we have also performed a set of experiments where the previous assays are repeated (with samples of 20, 10, 5, 2, and 1 spheroids and 2 × 10^5^, 1 × 10^5^, 5 × 10^4^, 2.5 × 10^4^, 1 × 10^4^, and 5 × 10^3^ cells in suspension and with successively increasing amounts of background activity levels) until no cell deposits are visible at PET scan. This set of experiments was performed once in triplicate.

### PET image quantification and data analysis

In the reconstructed PET images, 0.4 cm^3^ volumes of interests (VOI), located on the tip of each Eppendorf tube as well as in the background close to each tube, were defined. The total activity within each VOI was scored and corrected for Ga68 decay from administration. PET image contrast-to-noise ratio (CNR) for each cellular deposit was then determined using Eq. 1

1 $$ {\mathrm{CNR}}_j=\left|\frac{S_{\mathrm{c},\mathrm{j}}-{S}_{\mathrm{B}}}{S_{\mathrm{B}}}\right|\times \sqrt{n}\times \frac{S_{\mathrm{B}}}{\sigma_{\mathrm{B}}}=\left|\frac{S_{\mathrm{c},\mathrm{j}}-{S}_{\mathrm{B}}}{\sigma_{\mathrm{B}}}\right|\times \sqrt{n} $$

where *S*_c,j_ and *S*_B_ are the PET-derived activity concentration in the cell deposit “*j*” and in the background, respectively, σ_B_ is the background standard deviation, and *n* is the number of pixels in the VOI [20]. This equation is basically the signal-to-background noise ratio multiplied by the square root of the number of pixels in the VOI. The Rose criterion [21] for successful image lesion detectability, CNR > 3, was then used for binary classification of the PET images. A binomial logistic regression function (Eq. 2) was then used to determine the detection probability (DP) of Ga68-PSMA-11-PET/CT as a function of the number of living LNCaP cells in the deposit.

2 $$ DP=\frac{1}{1+{e}^{-\left({\beta}_0+{\beta}_1x\right)\kern0.5em }} $$

Where *β*_0_ and *β*_1_ are the regression coefficients.

The actual activity in each Eppendorf tube (decay corrected from administration) was also measured in an automated gamma counter (Wizard 2480, Perkin-Elmer). For each tube, a recovery coefficient (RC) was obtained as the ratio between the activity measured with the PET camera and with the gamma counter.

### The effect of Ga68-PSMA-11 mass concentration on specific binding

To investigate the Ga68-PSMA-11 mass concentration effect on specific binding to LNCaP cells, a saturation binding assay with increasing concentrations of the radioligand was performed. Samples of 1 × 10^5^ and 2 × 10^5^ single LNCaP cells in suspension where incubated at different concentrations of Ga68-PSMA-11 (range 2.8–338 nM) for 30 min at 37 °C. Pellet formation and supernatant removal was done as previously explained, and the activity in each tube was measured with the automated gamma counter. This experiment was also repeated with LNCaP cells seeded in 48 well-plates (1 × 10^5^ cells/well). After incubation, the supernatant was removed by aspiration and the wells washed three times with cold PBS. Cells were collected after trypsin treatment, and the activity measured in the automated gamma counter. The obtained results were all decay corrected and normalized to the SRA at administration time, and the results expressed as radioligand concentration per 100,000 cells (fmol/1 × 10^5^ cells). Non-specific binding was obtained from two competitive homologous binding assays with non-radioactive Ga68-PSMA-11. Two Eppendorf tubes with LNCaP cells in suspension were incubated for 30 min at 37 °C with 500 kBq of radioligand (Ga68-PSMA-11) at 31 and 71 nM molar concentration in the presence of 92 and 623 nM molar concentration of non-radioactive Ga-PSMA-11, respectively. Thereafter, the tubes were centrifuged at 2780 rpm during 3 min. The excess activity and medium was aspirated, and the pellet washed two times with cold PBS in each test tube. The activity in each tube was measured with an automated gamma counter. The results were then corrected for activity decay and normalized to the radioligand SRA at administration time and the results expressed as radioligand concentration per 100,000 cells (fmol/1 × 10^5^ cells). From these results, the non-specific binding was then linearly extrapolated to all radioligand concentrations used in the saturation binding assay. The specific binding was then obtained as the total binding minus the non-specific binding. The total receptor number *B*_max_, and the ligand equilibrium dissociation constant *K*_D_, where then obtained using Levenberg–Marquardt nonlinear regression analysis of the specific binding data (MATLAB with curve fitting Toolbox, Release 2015a, The MathWorks, Inc., Natick, MA, USA) and the model Eq. 3.

3 $$ \mathrm{Specific}\ \mathrm{binding}=\frac{B_{\mathrm{max}}\ast x}{K_{\mathrm{D}}+x} $$

where x denotes the radioligand concentration.

## Results

Throughout this set of experiments, the Ga68-PSMA-11 had a radiochemical purity of approximately 99% and a mean SRA of 45 ± 14 MBq/nmol (due to changes in the yield of the Ga68 generator).

**Fig. 1:**
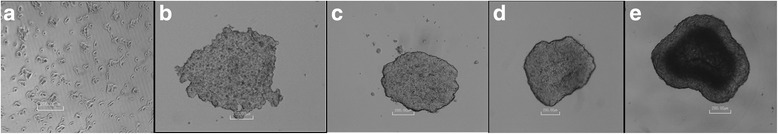
Time course for LNCaP spheroid formation from seeding (**a**) and at 24 h (**b**), 48 h (**c**), 72 h (**d**), and at 168 h (**e**). Final spheroid diameter of about 600 μm

Figure 1 shows an example of the formation course of an LNCaP spheroid used in this work. The number of living cells in each spheroid ranged between 3 × 10^3^ and 5 × 10^3^ cells at the different experiment occasions. PSMA cell expression was 99.8%. The FACS analysis on HCT116 cells showed a residual 0.6% PSMA expression.

### Detection limits of Ga68-PSMA-11-PET/CT

**Fig. 2:**
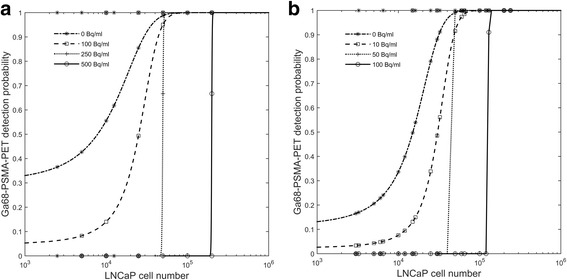
Ga68-PSMA-11-PET detection probability for LNCaP cells in suspension (**a**) or incubated as spheroids (**b**). The markers show the binary classification using the Rose criteria with CNR > 3 and the lines the binomial regression curves at different background levels

Figure 2a, b clearly shows that Ga68-PSMA-PET detection probability of a LNCaP cellular deposit decreases as the number of living cells in the deposit decreases and as the background activity level increases. This is a consequence of the combined effect of partial volume effects and spill in from the background activity in the object of interest. For cells incubated in suspension, *β*_1_ ranged from 0.0001 to 0.001 when changing the background activity from 0 to 500 Bq/ml. A similar change in *β*_1_ was obtained for spheroids but for a smaller increase in background activity from 0 to 100 Bq/ml. As Fig. 2a, b shows, the detection probabilities are significantly lower for spheroids as compared to suspension cells, reflecting the differences in biological characteristics. This also resulted in lower amounts of background required to reduce lesion visibility at PET.

Figure 2a reveals that the minimum detectable number of LNCaP cells (MDC) incubated in suspension for which PET detection probability is just 50% is 8 × 10^3^, 2.7 × 10^4^, 5 × 10^4^, and 1 × 10^5^ at 0, 100, 250, and 500 Bq/ml background levels, respectively. The MDC for LNCaP cells incubated as spheroids (Fig. 2b), is reached at higher cellular number 1.52 × 10^3^, 3.2 × 10^4^, 5 × 10^4^, and 1.3 × 10^5^, but at lower background levels of 0, 10, 50, and 100 Bq/ml, respectively. Extrapolating linearly these results to a background level of 1 kBq/ml resulted in a MDC at about 4 × 10^5^ and 1.1 × 10^6^ cells, for suspension and spheroids, respectively.

Without background activity, more than 90% PET detection probability was achieved at about 3 × 10^4^ LNCaP cells, for both suspension and spheroids.

**Fig. 3:**
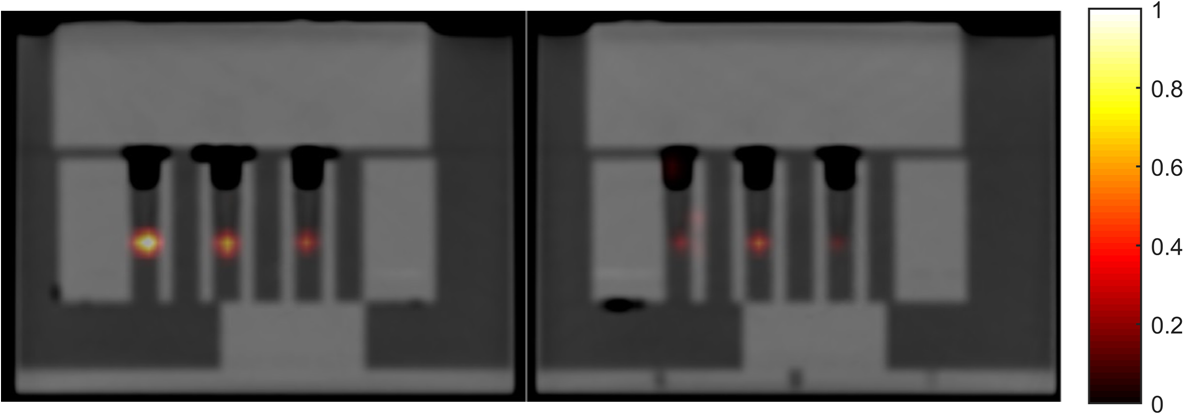
Ga68-PSMA-11-PET/CT image of different Eppendorf tubes all containing a pellet with 2 × 10^5^ HCT116+LNCaP cells. From left to right, the relative amount of LNCaP cells in the pellets is 100, 50, 25, 12.5, 5, and 2.5%, respectively

As an example of the relation between PCa cell number and PET detectability, Fig. 3 shows the coronal section of the water phantom containing the Eppendorf tubes with different numbers of LNCaP cells disseminated in pellets containing a total 2 × 10^5^ HCT116+LNCaP cells. This figure clearly shows a decline in PET signal as the number of LNCaP cells in the mixture decreases.

### Quantitative characteristics of Ga68-PSMA-11-PET/CT imaging

**Fig. 4:**
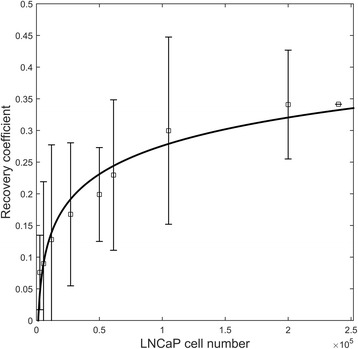
Recovery coefficient of Ga68-PSMA-11-PET/CT image as a function of number of LNCaP cells disseminated in a pellet of 2 × 10^5^ cells (HCT116+LNCaP). The actual activity uptake in the cells was obtained with an automated gamma counter. The error bars indicate standard error

Figure 4 illustrates the PET image RC as a function of LNCaP cell number. Displayed is also a logarithmic model function *y* = *a* + b*log(*x*), with regression coefficients *a* = − 0.4672 and *b* = 0.0645. The model predicts convergence to RC = 1 at 7.5 × 10^9^ LnCaP cells which may correspond to a cell deposit of around 7 cm^3^ [22].

**Fig. 5:**
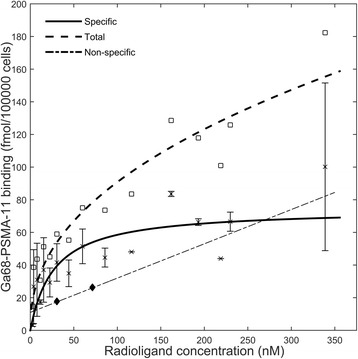
Total and specific saturation binding of Ga68-PSMA-11 to human LNCaP cells at equilibrium. The error bars indicate standard error

Figure 5 presents the results of the saturation of specific binding of Ga68-PSMA-11 in human LNCaP cells due to increasing radioligand concentration in the test-tubes. The estimated coefficients for the specific binding (with 95% confidence bounds) were *K*_D_ = 27.05 nM (− 1.213, 55.3) and *B*_max_ = 74.32 fmol/1 × 10^5^ cells (53.8, 94.85), R-square: 0.65. Figure 5 reveals the characteristic Ga68-PSMA-11-concentration response curve, which must be carefully considered in inter-individual comparative studies.

**Fig. 6:**
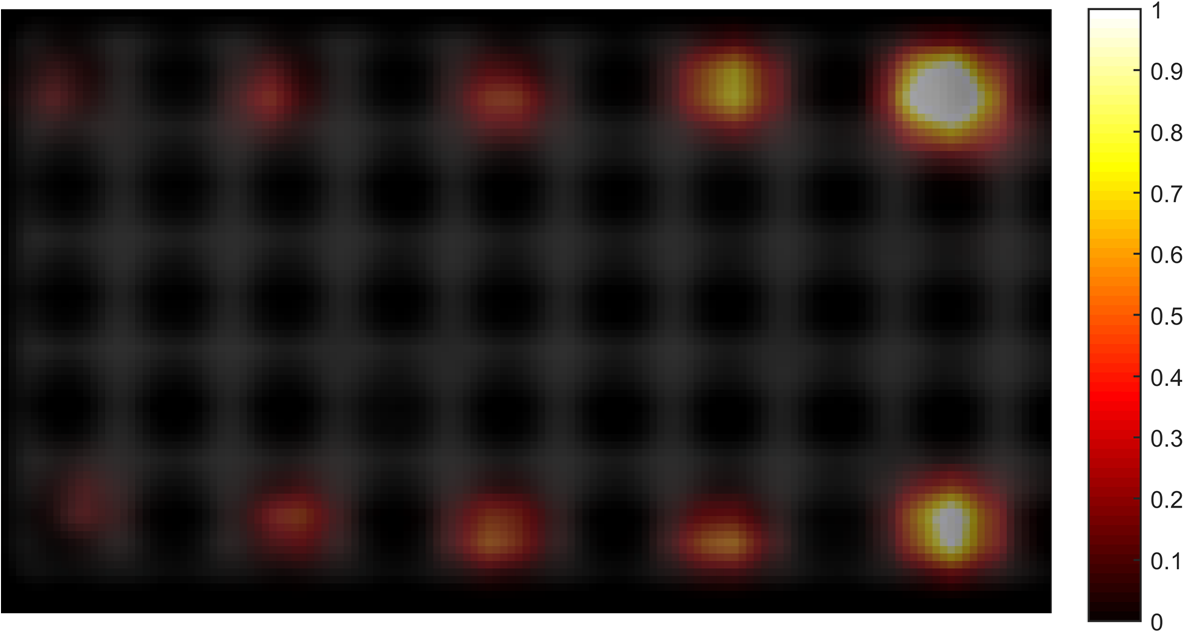
PET/CT image of 10 different Eppendorf tubes, all containing 1 × 10^5^ LNCaP cells in 1 ml media and incubated at different concentrations of Ga68-PSMA-11. First row of tubes from left to right, 193, 338, 544, 780, and 996 nM radioligand concentration, respectively (replicated in the second row)

As an example of the saturation effect on radioligand binding, Fig. 6 shows a PET/CT transversal section of a phantom containing five Eppendorf tubes (in duplicate) with the same number of LNCaP cells in each tube (1 × 10^5^ cells) but incubated at different concentrations of Ga68-PSMA-11 of 193, 338, 544, 780, and 996 nM, respectively.

## Discussions

In this study, the hallmarks of PCa imaging with Ga68-PSMA-11-PET/CT regarding detection limits and quantitative properties were stablished using a whole-body human PET/CT scanner and a well chamber. The theoretical MDC was in the order of 1 × 10^4^ LNCaP cells mixed in a cellular pellet of around 1 mm diameter with non-PSMA-expressing HCT cells and in the absence of background activity. At 1 kBq/ml background (which approximately mimics the activity concentration in a 70 Kg patient, 60 min after treatment with 140 MBq of Ga68-PSMA-11, 2 MBq/Kg), the MDC with PET increases then at a level between 4 × 10^5^ for LnCaP cells incubated as suspension and 1 × 10^6^ for LNCaP cells incubated as spheroids. However, this result may even further vary depending on the level of PSMA target expression, the SRA of the radioligand, and the used CNR cutoff value. A tumor containing these small amounts of LnCaP cells (22) is far smaller than the spatial resolution of the PET scanner. These results are not intuitive and demonstrate that the sensitivity of the PET scanner clearly overcomes the PET-limited spatial resolution, allowing to image objects well below the PET scanner intrinsic resolution. Figure 2b shows slightly lower detection probability levels for the same number of LNCaP cells as compared to Fig. 2a. This most likely reflect the in vivo situation as cellular spheroids attain most of the biological characteristics that tumor tissues have in vivo [23]. The MDC derived for 50% detection probability may realistically mimic the microscopic stage of the disease, which results in low levels of serum PSA. Interestingly, similar PET detection limit have been reported in clinical trials with patients with low PSA levels between 0.2 and 0.49 ng/ml [24]. Moreover, Figs. 2a, b also reveal that for cell deposits with more than 1 × 10^6^ cells, the sensitivity of Ga68-PSMA-11-PET in identifying small PCa cellular deposits is, against general perception, not coupled to the size of the lesion (or the nodal metastasis) [25] but rather to the density of PSMA-expressing cells mixed in the deposit. Lesion size, or rather the spread of PSMA-expressing PCa cells, only influences the quantification of the uptake due to partial volume effects (Fig. 4). The ability of Ga68-PSMA-11-PET/CT to image lesions containing a small number of cells, clearly challenge radical treatment. Hence the possibility of labelling PSMA with In111 together with a gamma-prove for radio-guided surgery should be considered [26].

In inter-individual or multicenter PCa PET/CT studies, it is important to consider the quantification characteristics of Ga68-PSMA-11-PET/CT imaging. Activity levels in the lesion depend on the level of prostate antigen expression, the cellular density, the PSMA membrane target affinity, the SAR of the Ga68-PSMA-11 at administration, the level of uptake in normal tissue expressing also PSMA targets, and finally non-specific binding. This is clearly demonstrated in Figs. 5 and 6 where radioligand binding is not linearly correlated with the concentration of the administered radioligand. This Ga68-PSMA-11 mass effect on PET image contrast can clearly jeopardize both tumor identification and quantification in prospective trials. Further, in the clinical settings, where a single Ga68-PSMA-11 batch is shared among several patients, the declining SRA could affect detection accuracy as increasing amounts of PSMA mass must be administered to keep the same level of administered Ga68-activity and image noise levels (usually 2 to 3 MBq per kg of body weight). As Fig. 5 shows, the dissociation constant of Ga68-PSMA-11 is low (27 nM) resulting in a high binding affinity. When the SRA decreases, the increasing mass of the radioligand may occupy an appreciable number of cell targets for the same PET signal, resulting in a misleadingly low binding potential (BP). Hence, since Ga68-PSMA-11 uptake depends on receptor density and SAR rather than just available activity, the use of standardized uptake values (SUV) should be precluded in quantitative studies. Scoring binding potential, which depends on the available receptor density and the SRA, may be more reproducible and robust than SUV, but it requires a dynamic PET scan with plasma sampling. This is generally not possible in routine clinical work where both camera time per patient and radioligand are limited. Therefore, in the clinical setting, the quantification of Ga68-PSMA-11 uptake in a tumor, should be carried relative to some reference organ or tissue and normalized to the actual SAR at administration. Which appropriate reference organ or tissue should be used is subject to further analysis.

### Limitations of this study

The results presented in this study are limited to LNCaP cell lines expressing high levels of PSMA receptors. The reproducibility of these results for other PCa cell lines with different PSMA target expression should also be investigated.

Although spheroids mimic some of the biological characteristics of tumors cells in tissues, the radioligand binding affinity may still be slightly different in vivo as tumors are not incubated with the tracer. However, these experiments cannot be reproduced in mice with xenografts, because the number of PCa cells and PSMA expression in the growing xenograft cannot be easily controlled and measured among different mice. Furthermore, the implanted tumor cells may even harbor different clones and/or develop tumor heterogeneity.

The measured non-specific binding (Fig. 5) includes both the radioligand trapped to the lipid membrane and to other membrane proteins and possible binding to the plastic of Eppendorf tubes. This slightly overestimated the non-specific binding curve obtained with the automated well chamber.

## Conclusions

Ga68-PSMA-11 binds to LNCaP cell membrane receptors with a high affinity. This results in high PET/CT detectability at low cell number in deposits much smaller than the PET system spatial resolution, even when these cells are mixed in a population of non-PSMA-expressing cells. This information is an essential hallmark for the individualization of patient management based on Ga68-PSMA-11-PET/CT and is one of the key issues in early detection of recurrent disease. The information provided in this work together with clinically available information could be merged in a monogram to determine the best therapeutic approach for every patient.

## References

[1] Babaian RJ, Troncoso P, Steelhammer LC, Lloreta-Trull J, Ramirez EI (1995). Tumor volume and prostate specific antigen: implications for early detection and defining a window of curability. J Urol.

[2] Blackwell KL, Bostwick DG, Myers RP, Zincke H, Oesterling JE (1994). Combining prostate specific antigen with cancer and gland volume to predict more reliably pathological stage: the influence of prostate specific antigen cancer density. J Urol.

[3] Bostwick DG, Graham SD, Napalkov P, Abrahamsson PA, Sant’Agnese PA, Algaba F (1993). Staging of early prostate cancer: a proposed tumor volume-based prognostic index. Urology.

[4] Kato RB, Srougi V, Salvadori FA, Ayres PPMR, Leite KM, Srougi M (2008). Pretreatment tumor volume estimation based on Total serum PSA in patients with localized prostate cancer. Clinics (Sao Paulo, Brazil).

[5] Tendulkar RD, Agrawal S, Gao T, Efstathiou JA, Pisansky TM, Michalski JM, Koontz BF, Hamstra DA, Feng FY, Liauw SL, Abramowitz MC, Pollack A, Anscher MS, Moghanaki D, Den RB, Stephans KL, Zietman AL, Lee WR, Kattan MW, Stephenson AJ (2016). Contemporary update of a multi-institutional predictive nomogram for salvage radiotherapy after radical prostatectomy. J Clin Oncol.

[6] Cortés-González JR, Castellanos E, Sandberg K, Eriksson MH, Wiklund P, Carlsson S, Cohn-Cedermark G, Harmenberg U, Gustafsson O, Levitt SH, Lennernäs B, Brandberg Y, Márquez M, Kälkner KM, Nilsson S (2013). Early salvage radiation therapy combined with short-term hormonal therapy in recurrent prostate cancer after radical prostatectomy: single-institution 4-year data on outcome, toxicity, health-related quality of life and co-morbidities from 184 consecutive patients treated with 70 Gy. Int J Oncol.

[7] Hovels AM, Heesakkers RA, Adang EM, Jager GJ, Strum S, Hoovegeveen YL, Severens JL, Barentsz (2008). The diagnostic accuracy of CT and MRI in the staging of pelvic lymph nodes in patients with prostate cancer: a meta-analysis. Clin Radiol.

[8] Picchio M, Piert M (2013). Prostate cancer imaging. Eur J Nucl Med Mol Imaging.

[9] Meyer C, Ma B, Kunju LP, Davenport M, Piert M (2013). Challenges in accurate registration of 3-D medical imaging and histopathology in primary prostate cancer. Eur J Nucl Med Mol Imaging.

[10] Afshar-Oromieh A, Malcher A, Eder M, Eisenhut M, Linhart HG, Hadaschik BA (2013). PET imaging with a [68Ga]gallium-labelled PSMA ligand for the diagnosis of prostate cancer: biodistribution in humans and first evaluation of tumour lesions. Eur J Nucl Med Mol Imaging.

[11] Afshar-Oromieh A, Holland-Letz T, Giesel FL, Kratochwil C, Mier W, Haufe S, Debus N, Eder M, Eisenhut M, Schäfer M, Neels O, Hohenfellner M, Kopka K, Kauczor HU, Debus J, Haberkorn U (2017). Diagnostic performance of (68)Ga-PSMA-11 (HBED-CC) PET/CT in patients with recurrent prostate cancer: evaluation in 1007 patients. Eur J Nucl Med Mol Imaging.

[12] Schwenck J, Rempp H, Reischl G, Kruck S, Stenzl A, Nikolaou K, Pfannenberg C, la Fougère C (2017). Comparison of (68)Ga-labelled PSMA-11 and (11)C-choline in the detection of prostate cancer metastases by PET/CT. Eur J Nucl Med Mol Imaging.

[13] Bluemel C, Linke F, Herrmann K (2016). Impact of 68Ga-PSMA PET/CT on salvage radiotherapy planning in patients with prostate cancer and persisting PSA values or biochemical relapse after prostatectomy. EJNMMI Res.

[14] Leiblich A, Stevens D, Sooriakumaran P (2016). The utility of molecular imaging in prostate cancer. Curr Urol Rep.

[15] Sanchez-Crespo A, Andreo P, Larsson S (2004). Positron flight in human tissues and its influence on PET image spatial resolution. Euro J Nuc Med Molec Imaging.

[16] Sanchez-Crespo A, Larsson SP (2006). The influence of photon depth of interaction and non-collinear spread of annihilation photons on PET image spatial resolution. Eur J Nucl Med Mol Imaging.

[17] Sanchez-Crespo A (2013). Comparison of gallium-68 and fluorine-18 imaging characteristics in positron emission tomography. Appl Radiat Isot.

[18] Eder M, Neels O, Müller M, Bauder-Wüst U, Remde Y, Schäfer M, Hennrich U, Eisenhut M, Afshar-Oromieh A, Haberkorn U, Kopka K (2014). Novel preclinical and radiopharmaceutical aspects of [68Ga]Ga-PSMA-HBED-CC: a new PET tracer for imaging of prostate cancer. Pharmaceuticals (Basel).

[19] Herrmann R, Fayad W, Schwarz S, Berndtsson M, Linder S (2008). Screening for compounds that induce apoptosis of cancer cells grown as multicellular spheroids. J Biomol Screen.

[20] Cherry SR, Sorenson JA, Phelps ME (2012). Physics in nuclear medicine.

[21] Rose A (1973). Vision: human and electronic.

[22] Del Monte U (2009). Does the cell number 10(9) still really fit one gram of tumor tissue?. Cell Cycle.

[23] Takagi A, Watanabe M, Ishii Y, Morita J, Hirokawa Y, Matsuzaki T, Shiraishi T (2007). Three-dimensional cellular spheroid formation provides human prostate tumor cells with tissue-like features. Anticancer Res.

[24] von Eyben FE, Picchio M, von Eyben R, Rhee H, Bauman G (2016). (68)Ga-labeled prostate-specific membrane antigen ligand positron emission tomography/computed tomography for prostate cancer: a systematic review and meta-analysis. Eur Urol Focus.

[25] Jilg CA, Drendel V, Rischke HC, Beck T, Vach W, Schaal K, Wetterauer U, Schultze-Seemann W, Meyer PT (2017). Diagnostic accuracy of Ga-68-HBED-CC-PSMA-ligand-PET/CT before salvage lymph node dissection for recurrent prostate cancer. Theranostics.

[26] Rauscher I, Düwel C, Wirtz M, Schottelius M, Wester HJ, Schwamborn K, Haller B, Schwaiger M, Gschwend JE, Eiber M, Maurer T (2017). Value of (111) in-prostate-specific membrane antigen (PSMA)-radioguided surgery for salvage lymphadenectomy in recurrent prostate cancer: correlation with histopathology and clinical follow-up. BJU Int.

